# Health-related quality of life issues, including symptoms, in patients with active COVID-19 or post COVID-19; a systematic literature review

**DOI:** 10.1007/s11136-021-02908-z

**Published:** 2021-06-19

**Authors:** Cecilie Delphin Amdal, Madeline Pe, Ragnhild Sørum Falk, Claire Piccinin, Andrew Bottomley, Juan Ignacio Arraras, Anne Sophie Darlington, Kristin Hofsø, Bernard Holzner, Nina Marie Høyning Jørgensen, Dagmara Kulis, Stein Arne Rimehaug, Susanne Singer, Katherine Taylor, Sally Wheelwright, Kristin Bjordal

**Affiliations:** 1 Research Support Services, Oslo University Hospital, Sogn Arena, Nydalen, Post Box 4950, Oslo, Norway; 2 Department of Oncology, Oslo University Hospital, Oslo, Norway; 3 Quality of Life Department, EORTC, Brussels, Belgium; 4 Servicio de Navarro de Salud, Pamplona, Spain; 5 School of Health Sciences, University of Southampton, Southampton, UK; 6 Department of Research and Development, Oslo University Hospital, Oslo, Norway; 7 Lovisenberg Diaconal University College, Oslo, Norway; 8 University Hospital of Psychiatry II, Medical University of Innsbruck, Innsbruck, Austria; 9 Medical Library at Oslo University Hospital, University of Oslo Library, Oslo, Norway; 10 Sunnaas Rehabilitation Hospital, Nesoddtangen, Norway; 11 Institute of Medical Biostatistics, Epidemiology and Informatics, University Medical Centre of Johannes Gutenberg University Mainz, Mainz, Germany; 12 Health Sciences, University of Southampton, Southampton, UK; 13 Faculty of Medicine, University of Oslo, Oslo, Norway

**Keywords:** COVID-19, Health-related quality of life, Symptoms, Concerns, Patient-reported outcome

## Abstract

**Purpose:**

This systematic review was performed to identify all relevant health-related quality of life (HRQoL) issues associated with COVID-19.

**Methods:**

A systematic literature search was undertaken in April 2020. In four teams of three reviewers each, all abstracts were independently reviewed for inclusion by two reviewers. Using a pre-defined checklist of 93 criteria for each publication, data extraction was performed independently by two reviewers and subsequently compared and discussed. If necessary, a third reviewer resolved any discrepancies. The search was updated in February 2021 to retrieve new publications on HRQoL issues including issues related to the long-term consequences of COVID-19.

**Results:**

The search in April 2020 identified 3342 potentially relevant publications, and 339 publications were selected for full-text review and data extraction. We identified 75 distinct symptoms and other HRQoL issues categorized into 12 thematic areas; from general symptoms such as fever, myalgia, and fatigue, to neurological and psychological issues. The updated search revealed three extra issues experienced during active disease and long-term problems with fatigue, psychological issues and impaired cognitive function.

**Conclusion:**

This first comprehensive systematic review provides a detailed overview of the wide range of HRQoL issues experienced by patients with COVID-19 throughout the course of the disease. It demonstrates the devastating impact of the disease and provides critically important information for clinicians, to enable them to better recognize the disease and to provide knowledge important for treatment and follow-up. The results provided the foundation for the international development of a COVID-19 specific patient-reported HRQoL questionnaire.

**Supplementary Information:**

The online version contains supplementary material available at 10.1007/s11136-021-02908-z.

## Plain English summary

This literature review provides the first overview of the health-related quality of life issues experienced by patients with COVID-19, throughout the disease and in the early recovery phase. Although many scientific papers have reported on specific groups of symptoms, a full picture has been missing. In this systematic review, the results from studies in all continents demonstrate that patients with this disease experience a wide range of different symptoms and concerns over the course of the disease. Patients with symptoms from Covid-19 commonly presented with fever, muscle pain, cough, shortness of breath and diarrhoea. Rare symptoms such as hair loss, skin problems, painful urination, nerve pain and tightness of the chest were also reported. Patients in rehabilitation after the acute COVID-19 period, experienced ongoing problems with extreme tiredness (fatigue), psychological issues and impaired mental function. Most organ systems can be affected, and the disease may have a devastating impact on the patients’ health-related quality of life. COVID-19 affects people of all ages, and the symptom burden may also give reduced working abilities and societal challenges. This important information can aid clinicians to better recognize the disease and provides important knowledge for treatment and follow-up. The results will be used in the development of a questionnaire assessing these issues in future patient populations. For patients with COVID-19 and their next of kin, this will provide greater insight into many different clinical aspects of the disease and may support self-management.

## Introduction

At the end of 2019, a cluster of pneumonia caused by a new beta-coronavirus, SARS coronavirus 2 (SARS-CoV-2), was reported in Wuhan, Hubei Province, China [1]. As this coronavirus-induced disease (COVID-19) has recently emerged, it is of paramount importance to get a systematic overview of the patient’s experiences related to the disease trajectory. As potential therapeutics and vaccines has been developed and tested, it is increasingly important to understand the virus’ burden and its impact on health-related quality of life (HRQoL).

HRQoL is usually defined as a multidimensional concept [2]. It is subjective and best assessed by the patients themselves using patient-reported outcome measures (PROMs). Questionnaires which have been created to quantify HRQoL usually include measurements of the patient’s physical, psychological and social functioning, in addition to symptoms of disease and treatment [3]. In the first month of the pandemic, systematic reports on patient-reported outcomes of the COVID-19 were scarce. Although, there were increasing numbers of case histories and individual descriptions of patient experiences demonstrating the devastating nature of the disease, there were no COVID-19 specific questionnaires available and researchers who wanted to include PROMs in their studies had to settle for generic instruments or symptom checklist [4, 5].

Understanding the issues that COVID-19 patients face during various stages of the disease may inform diagnosis and prognosis, and provide important indicators of the benefits and risks associated with treatment. Such information may eventually help to identify stages of disease progression and may be used to guide clinical practice and inform the measurement of outcomes in therapeutic research. Given that patient-reported results were largely missing, in order to gather information regarding the impact of the disease on patients’ HRQoL, observer-rated or non-systematic reports on patients’ symptoms, functioning and concerns were used as an initial surrogate.

From the early stages of the pandemic, it was recognized that the clinical manifestation of COVD-19 varies considerably and the effect of disease and treatment on patients’ HRQoL in the short and long term is still not fully understood. Older adults and individuals with underlying conditions such as diabetes, obesity, and heart and lung disease have been shown to be particularly at risk for developing severe forms of illness [6] and disease progression occurs significantly more rapidly in elderly people compared to the younger population [7]. Symptomatology may evolve considerably during the acute and sub-acute phases of disease, with some symptoms emerging only weeks after the diagnosis [8]. The most severe cases of COVID-19 are prone to a variety of complications, including acute respiratory distress syndrome (ARDS), secondary infections, and acute heart injury [9]. The highly contagious nature of the disease makes the experience of COVID-19 particularly isolating for patients, many of whom must remain in strict confinement at home or in clinics, with little to no direct contact with loved ones and often limited support. This added psychosocial burden may exacerbate an already difficult situation [10]. Patients with confirmed COVID-19 may experience anxiety and fear concerning the high risk for contagion [11] along with guilt and depression in cases of known transmission. In addition to the broader psychosocial implications of a COVID-19 diagnosis, the experience of the disease and its treatment and symptoms may result in depression, anxiety, impaired memory, insomnia, post-traumatic stress disorder (PTSD), and other serious psychiatric complications [12]. Insight into the symptoms and issues that patients who have been diagnosed with COVID-19 face, along with their potential impact on functioning and patients’ HRQoL, is an important step in understanding the clinical manifestations and their relationship to underlying biological mechanisms and disease prognosis.

With this project, the authors aimed to identify the HRQoL issues relevant for patients with COVID-19, and to use the results as a starting point for the development of a disease-specific patient-reported outcome measure (PROM) for future studies in this patient group. This comprehensive systematic review was carried out to obtain broad coverage of all possible issues to determine the range and prevalence of HRQoL issues affecting COVID-19 patients, relating to their disease and treatment. We explored issues reported by patients, as well as those observed by healthcare providers and other proxy-reporters for patients with COVID-19 during short- and long-term follow-up.

## Methods

The review question was to understand what symptoms and other HRQoL issues are reported by patients with COVID-19. The Cochrane guidelines for Systematic Reviews [13] and PRISMA guidelines were followed (see online Appendix 1, for the PRISMA checklist) [14]. The review was registered in PROSPERO (ID = CRD42020185995) [15]. This review differs from a standard systematic review as all report types were included, irrespective of quality, to ensure the broadest coverage possible of COVID-19 patients’ symptoms, functioning and concerns.

### Search strategy and selection criteria

**Fig. 1:**
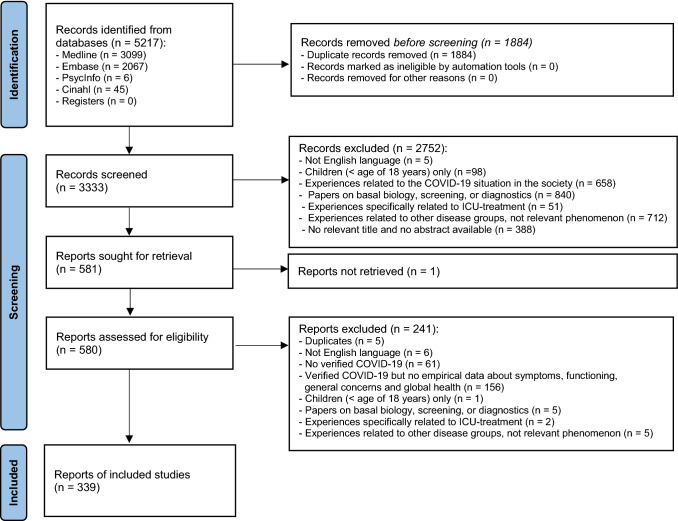
PRISMA Flow chart of eligibility screening and inclusion

A literature search was performed using a combination of controlled vocabulary (e.g. MeSH, EmTree-terms) and free-text search terms for COVID-19, coronavirus disease and related symptoms and concerns, quality of life and patient-reported outcomes. The searches were conducted in Embase, MEDLINE, PsycInfo and CINAHL on April 28th 2020. No filters for study design were applied. Only articles published in English, between January 2019 and April 2020 were included. The PRISMA flow diagram (Fig. 1) was used to present the different stages of the review process [16]. The complete search strategy is presented in online Appendix 2.

All references were screened by title and abstract if available. The inclusion criteria were: any publication, letter or news reports which included reports by patients, health care professionals or other proxy-reports of COVID-19 patients’ symptoms and other experienced HRQoL issues related to their disease and treatment, at diagnosis, during active disease and during late or long-term follow-up. Exclusion criteria were publications written in a language other than English, or limited to children under the age of 18 years, or publications limited to the societal impact of COVID-19, biological aspects of the virus, or papers specifically related to intensive care unit (ICU) treatment, experiences related to the effect of the pandemic on other diseases, or the emotional reactions of the professionals treating COVID-19 patients.

### Screening and full-text review

Before starting, a session to ensure consistency in the screening process was held. Rayyan QCRI® [17], a web application, was used to facilitate the screening publications. Reviewers were divided into four interdisciplinary, multicultural groups with three reviewers, including at least one clinician, in each group. Each reviewer participated in two pairings within their group and if needed acted as a consultant to the third pairing in the group. Both reviewers within each pair independently screened abstracts and resolved intra-pair disagreements through discussion or through consultation with the third reviewer in the group if consensus could not be reached. A similar process was used for the full-text papers of included abstracts: they were independently checked for eligibility by two reviewers who also extracted data using a checklist of 93 criteria (see below). Any disagreements were resolved through discussion and consultation with the third reviewer if necessary.

### Data extraction and categorization

The initial data extraction sheet aimed to include essential publication characteristics and to pre-specify as many symptoms and HRQoL issues as possible to facilitate data extraction. A review of randomly selected publications was performed until no new issues were retrieved (issue saturation, after 52 publications). The data extraction sheet was pilot-tested by all reviewers on ten randomly selected included studies. This resulted in 93 criteria, classified into four broad categories: (1) inclusion/exclusion criteria, (2) general information on the paper and patient characteristics, (3) reported COVID-19 patient issues, and (4) use of validated PRO questionnaires (online Appendix 3).

Papers were categorised according to study design: reviews, randomised clinical trials, observational studies with > 5 patients and case studies with ≤ 5. Reported issues were grouped into categories based on “descriptive themes” that capture HRQoL issues. Categorizing the codes based on descriptive themes was done through a consensus discussion within the project team. The stage of disease at the time the issue reportedly started was categorized as 1: before, at or shortly after diagnosis, 2: additional symptoms starting after hospitalization or during active disease, and 3: late or long-term (defined as starting after the isolation period ended). Data were analysed using a narrative synthesis. Patient experience and perspective about their HRQoL issues were extracted.

### Statistical analyses

Descriptive statistics are presented using absolute numbers and percentages, and stratified by study type. The descriptions of symptoms, concerns, and functioning were based on observational studies only. Review papers were excluded in the descriptive statistics to avoid double counting of issues. Issues were included if reported in more than one paper or in one paper with more than 10 patients. Comparison of the presence of issues in males and females, and in young (< 41 years) and elderly (> 70 years) patients was given as an absolute difference in proportions. Graphically, the cumulative proportion by time of publication was presented for selected issues to illustrate different trends in reporting of symptoms during the first four months after the first publication on 24th January 2020.

### Updated literature searches

Updated simplified literature searches were performed on 29th October 2020 and 2nd February 2021, due to the time lag from the original literature search and the large amount of literature produced for this disease. The searches were performed in MEDLINE (search strategies, online Appendix 4). The first author screened references using the same inclusion and exclusion criteria as described for the main search. Full-text papers were reviewed for issues not already identified, reports on long-term effects after COVID-19, and also for the inclusion of PROMs.

## Results

The titles and abstracts of 3333 publications were screened and reasons for ineligibility were documented for each of the 2752 papers not selected for full-text review (Fig. 1). We were unable to retrieve a single-page publication published in a magazine with unknown author. Of the 580 papers eligible for full-text review, a total of 339 papers were included (online Appendix 5, details included studies), and the reasons for exclusion of the other 241 papers are displayed in Fig. 1 and online Appendix 6. The main reasons for exclusions were “no empirical data about symptoms, functioning and concerns” and “no verified COVID-19 infection”.

### Description of included papers and patients

**Fig. 2:**
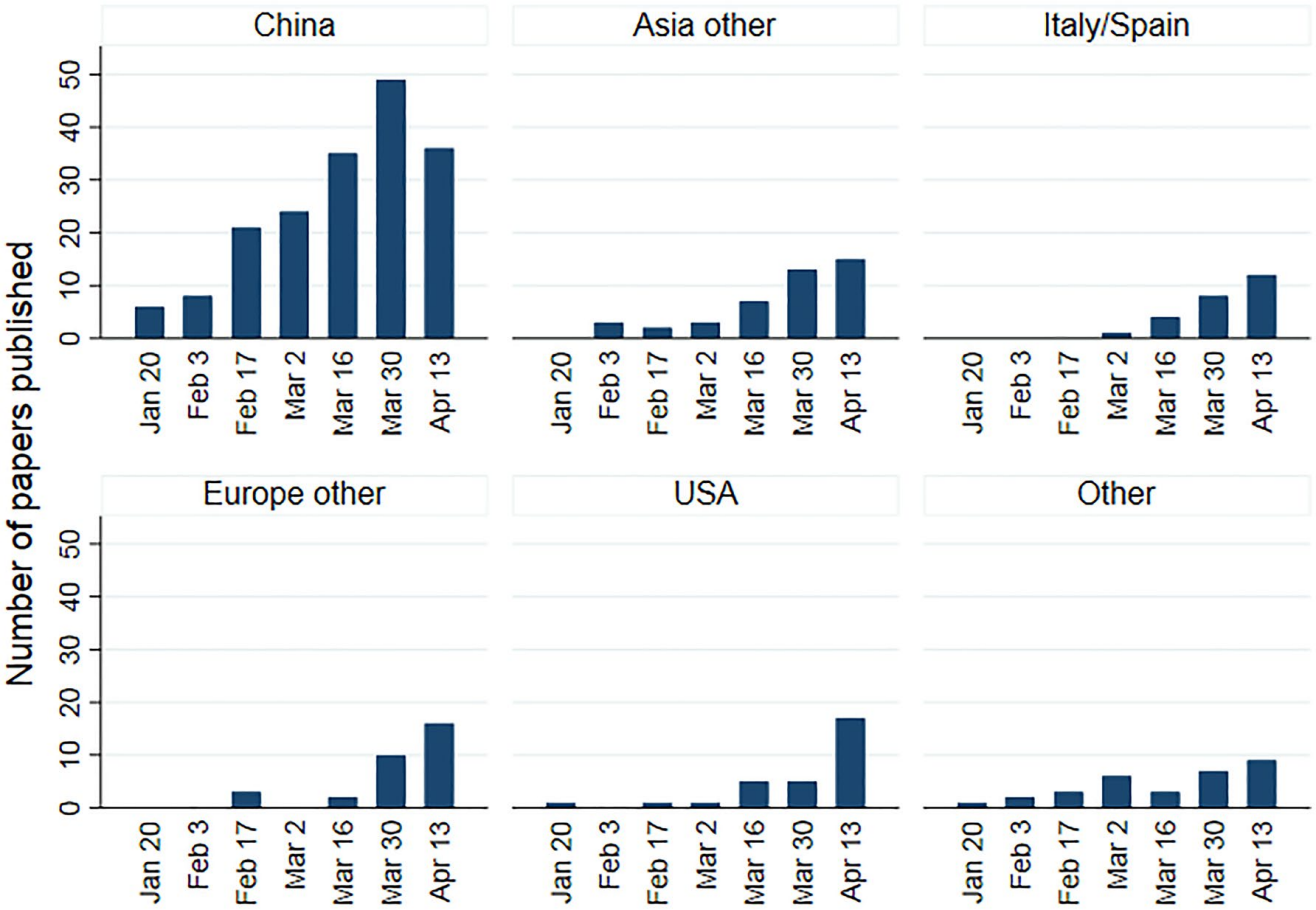
Number of published papers according to date of publication and geographical origin. Other: non-European countries and multiple countries

China was the first country to publish symptom reports after the outbreak of the pandemic and continued to be the dominant publication country, accounting for more than half of the 339 included papers throughout this first period from January to end of April 2020 (Table 1, Fig. 2).

**Table 1:** Description of papers included stratified by type of study, *n* = 339

		All *n* = 339		Reviews *n* = 34		Observational studies (> 5 patients) *n* = 159		Case studies (≤ 5 patients) *n* = 146	
		*n*	%	*n*	%	*n*	%	*n*	%
Country	China	179	53	16	47	111	70	52	36
	Asia other	43	13	0	0	10	6	33	23
	Italy/Spain	25	7	1	3	7	4	17	12
	Europe other	31	9	1	3	8	5	22	15
	USA	30	9	0	0	13	8	17	12
	Other non-European (including Australia, Africa and South America)	13	4	1	3	7	4	5	3
	Multiple countries	18	5	15	44	3	2	0	0
Type of report	PROMs included^a^	9	3	0	0	8	5	1	1
	PROMs not included	316	93	29	85	148	93	139	95
	*Unknown*	14	4	5	15	3	2	6	4
Sample size	Number of patients; median/range	12/1–58,663		1576/21–58,663		73/6–9282		1/1–5	
	*Not reported*	7	2	7	21	0	0	0	0

Numbers are frequencies and proportions if not otherwise specified

*PROMs* patient-reported outcome measures

^a^One psychological questionnaire, four related to taste and smell, and four of general symptom assessment

The first reports from other countries started a few weeks later with an increase in publication rate from the end of March 2020. Most studies, independent of origin were observational studies (Table 1). No randomized trials were identified. Nine publications included PROMs. In one study, the psychological impact was assessed with the Impact of Events Scale—Revised (IES-R) [18]. Taste and smell disturbances were assessed in four studies; one used the “Olfactory disorders, Short version of questionnaire of olfactory disorders negative statements of patient” [19], whilst the other three used unnamed ad hoc questionnaires. Four additional studies used unnamed ad hoc questionnaires for patient-reported general symptom assessment.

The number of patients ranged from one to more than fifty thousand per study (Table 1). Most reports included both male and females and all age groups above 18 years old were represented (Table 2). The comorbidity of the patients was either not present or not described in 37% of the publications. When comorbidities were described, hypertension, diabetes, and cardiovascular and pulmonary disease were most frequently reported (Table 2). Most patients were hospitalized, but several papers described symptoms and concerns of patients in nursing or isolation units or patients who were isolated within their own home.

**Table 2:** Description of patients included in the 339 papers stratified by type of study

		All papers *n* = 339		Review *n* = 34		Observational studies (> 5 patients) *n* = 159		Case studies (≤ 5 patients) *n* = 146	
		*n*	%	*n*	%	*n*	%	*n*	%
Gender	Male	65	19	0	0	1	0**.**6	64	44
	Female	51	15	1	3	6	4	44	30
	Both	204	60	25	74	145	91	34	23
	*Unknown*	19	6	8	24	7	4	4	3
Age Specified	Yes, young 18–40	211	67	16	67	131	86	63	45
	Yes, middle-aged 41–70	242	76	18	75	143	94	80	57
	Yes, elderly/old > 70	138	44	14	58	96	63	27	19
	* Not specified*	23	7	10	29	7	4	6	4
	Reported median (years)/range	49/22–86.5		50/41–70		48.8/22–86.5		49/22–81	
Comorbidity	Hypertension	139	41	15	44	91	57	33	23
	Diabetes	131	39	17	50	91	57	23	16
	Cardiovascular disease	110	32	12	35	80	50	18	12
	Pulmonary disease	104	31	11	32	85	53	8	5
	Cancer	67	20	7	21	52	33	8	5
	Kidney/urinary system disease	66	19	4	12	47	30	15	10
	Liver disease	61	18	7	21	47	30	7	5
	Cerebrovascular disease	33	10	1	3	31	20	1	0.7
	Autoimmune disease and immunodeficiency	33	10	1	3	20	13	12	8
	Endocrine disease/disorder	25	7	1	3	20	13	4	3
	Gastrointestinal disease/disorder	20	6	1	3	18	11	1	0.7
	Neurological disease	16	5	2	6	12	8	2	1
	Infectious disease	15	4	1	3	12	8	2	1
	Psychiatric disease	6	2	0	0	4	3	2	1
	Other specified	39	12	3	9	20	13	16	11
	*No/not described*	124	37	15	44	46	29	63	43
Hospitalisation	Intensive care unit (ICU)	100	29	15	44	59	37	26	18
	Hospital, not ICU	266	78	19	56	133	84	114	78
	Nursing home or isolation units or own home	49	14	3	9	23	14	23	16
	*No/not described*	45	13	15	44	17	11	13	9

Numbers are frequencies and proportions if not otherwise specified

### Symptoms, concerns, and functions reported

**Fig. 3:**
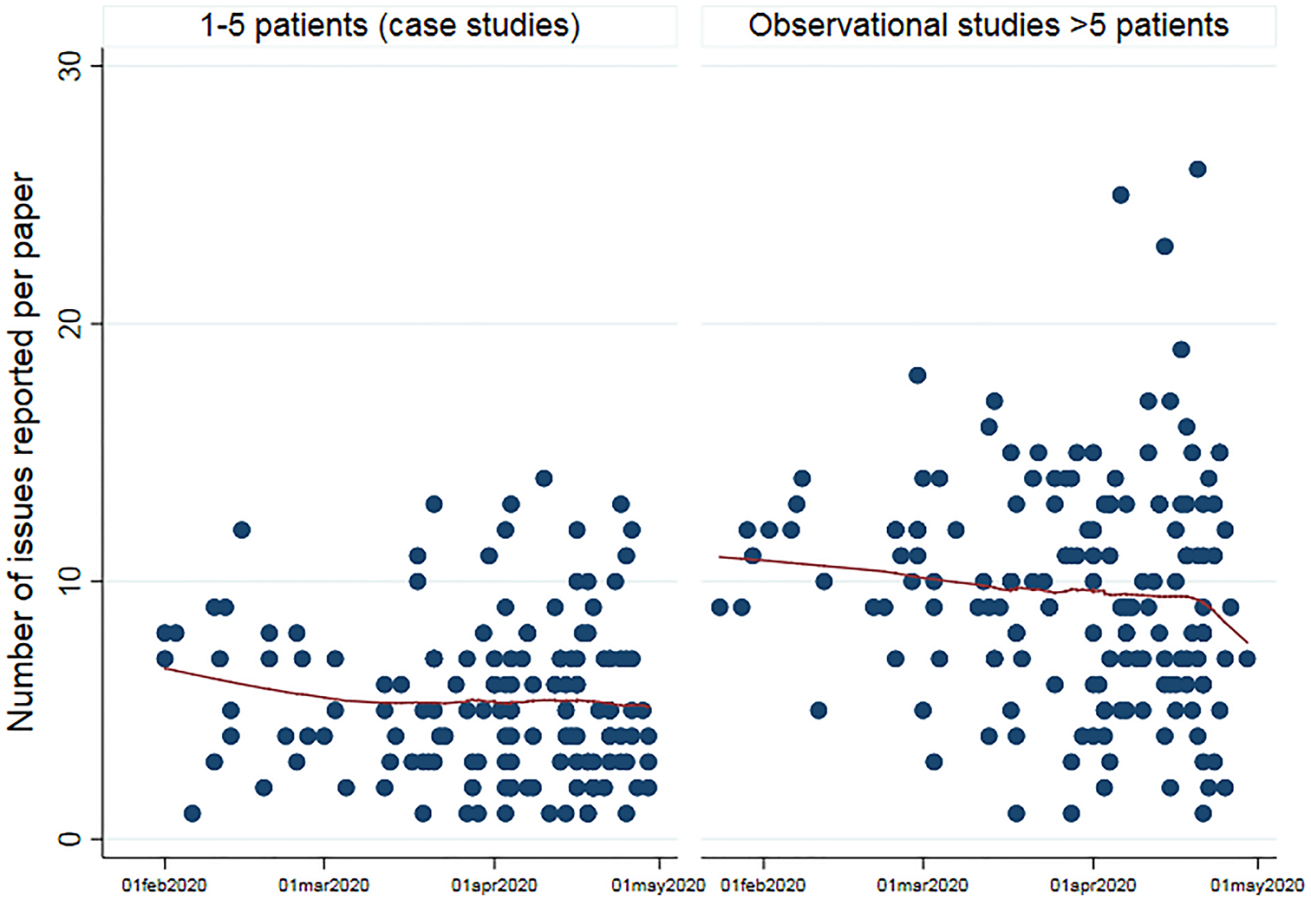
Number of issues reported per paper according to date of publication for observational studies. *Lines are local weighted average smoother with bandwidth 0.8*

In total, 95 different symptoms, concerns, and functions were described, of which 75 issues were reported in more than one paper or in one paper with more than 10 patients (Table 3). Amongst the observational studies, a median of seven issues (range 1–26) per publication was reported. The number of symptoms reported per paper was stable (median varied between 6 and 10) from January to end of April 2020, but some observational studies reported a higher number of issues at the end of the period compared to studies in the beginning of the period (Fig. 3).

**Table 3:** Description of symptoms, reduced functions and concerns from observational studies, 305 papers

Issue^a^	Number of papers (%)	Start of symptom early/active^b^	Descriptive themes
Fever	273 (90)	262/11	General symptoms
Chills or shivering	53 (17)	50/3	
Fatigue or asthenia including weakness	153 (50)	150/3	
Malaise/feeling sick	25 (8)	25/0	
Extensive sweating or night sweats	4 (1)	4/0	
Dizziness	32 (10)	31/1	
Drowsiness	5 (2)	4/1	Level of consciousness
Confusion or delirium	20 (7)	17/3	
Unconscious	3 (1)	3/0	
Cough	249 (82)	236/13	Respiration
Shortness of breath, dyspnoea or respiratory distress	196 (64)	178/18	
Expectoration lung	82 (27)	80/2	
Haemoptysis/blood in the expectoration	25 (8)	24/1	
Oral (mouth) mucus or saliva	13 (4)	13/0	Mouth/throat
Pharyngodynia/sore throat	113 (37)	110/3	
Throat congestion	2 (0,7)	2/0	
Tonsil swelling	1 (0,3)	1/0	
Sneezing	8 (3)	8/0	Nose
Mucus in the nose or nasal congestion	35 (11)	33/2	
Rhinorrhoea/coryza/runny nose	63 (21)	58/5	
Nasal symptoms	1 (0.3)	1/0	
Epistaxis/nose bleeding	2 (0.7)	2/0	
Otalgia/ear pain	4 (1)	4/0	Ear
Hearing loss	2 (0.7)	2/0	
Tinnitus	3 (1)	3/0	
Irritation eyes or sore eyes	9 (3)	8/1	Eye
Red eyes	6 (2)	6/0	
General pain	12 (4)	10/2	Pain
Myalgia/general muscle pain	135 (44)	132/3	
Neuropathic/neurological pain	1 (0,3)	1/0	
Headache	116 (38)	115/1	
Muscle soreness	1 (0.3)	2/0	
Arthralgia/ joint pain	15 (5)	15/0	
Back pain	3 (1)	3/0	
Facial pain or heaviness	1 (0.3)	1/0	
Loss of taste	21 (7)	20/1	Neuropathic–neurological
Loss of smell	23 (8)	21/2	
Sight problems or vision	2 (0.7)	2/0	
Uncoordinated movements	1 (0.3)	1/0	
Seizure	4 (1)	3/1	
Diarrhoea	152 (50)	140/12	Gastrointestinal
Constipation	7 (2)	6/1	
Nausea	73 (24)	70/3	
Vomiting	74 (24)	71/3	
Anorexia/loss of appetite	43 (14)	43/0	
Stomach ache/abdominal pain	50 (16)	47/3	
Belching	2 (0.7)	2/0	
Acid reflux	1 (0.3)	1/0	
Abdominal distention	1 (0.3)	1/0	
Bloating	2 (0.7)	2/0	
Tenesmus/bowel cramps	1 (0.3)	1/0	
Gastrointestinal discomfort	1 (0.3)	1/0	
Gastrointestinal symptoms	6 (2)	6/0	
Dysuria	1 (0.3)	1/0	Urinary
Haematuria/blood in urine	2 (0.7)	2/0	
Rash including urticaria	18 (6)	14/4	Skin
Pruritus/itching	6 (2)	5/1	
Skin pain or burning sensation	3 (1)	3/0	
Hair loss	1 (0.3)	1/0	
Chest pain	48 (16)	44/4	Chest—cardio
Tightness of chest	34 (11)	32/2	
Chest distress	13 (4)	12/1	
Heart palpitations	8 (3)	7/1	
Anxiety	9 (3)	6/3	Psychological–psychiatric
Depression	3 (1)	2/1	
Distress	2 (0.7)	1/1	
Tension	1 (0.3)	1/0	
Agitation	3 (1)	2/1	
Anger	2 (0.7)	2/0	
Insomnia	2 (0.7)	2/0	
Fear of being discriminated against society	2 (0.7)	2/0	Concern
Fear of future outcome	2 (0.7)	2/0	
Fear of cold	1 (0.3)	1/0	
Activity daily living	2 (0.7)	2/0	Functions
Social activities	1 (0.3)	1/0	

No papers reported additional symptoms specifically appearing at long-term follow-up

^a^Issues are included if reported in more than one paper or in one paper with more than 10 patients

^b^The stage of disease during which a symptom or issue were reported to appear was categorized as “early” (before, at or shortly after diagnosis) or “active” (additional symptoms starting after hospitalization or during active disease)

**Fig. 4:**
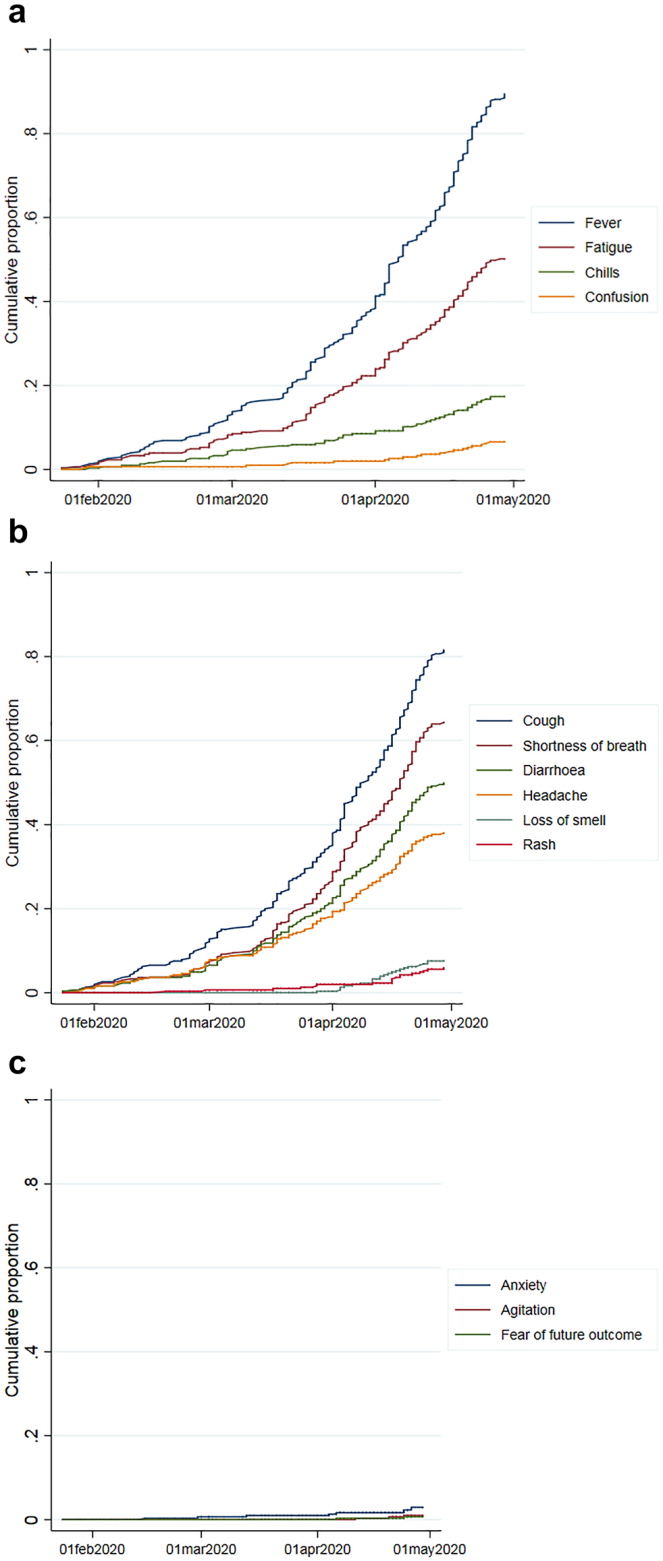
Cumulative proportion of papers over time reporting selected **a** general symptoms, **b** other symptoms and **c** psychological symptoms and concerns

From the start of the pandemic, general symptoms were reported: fever in 90% and fatigue in 50% of the publications (Table 3, Fig. 4a). Symptomatic patients commonly presented with cough, shortness of breath, myalgia, headache, and diarrhoea although the range and severity of symptoms differed considerably [8, 20]. Reports on patients experiencing loss of smell, chest pain, and skin problems came later with an increase from mid-March 2020 (Fig. 4b). All issues were reported to start near the time of diagnosis, and 33 also appeared later during active disease. Very few studies looked at the psychological aspects of the disease (Table 3, Fig. 4c). Some case reports described patients that reported concerns such as fear of being discriminated against in society and the fear of future outcomes [21, 22]. Only one study reported how symptoms affected patients’ ability to carry out social activities and influenced their HRQoL [19].

Most studies included patients of all age groups, 48 observational studies included only young patients (age 18–40 years) whilst 19 studies included only the elderly (age > 70 years) (Table 4). In the descriptive analyses, young patients reported more fatigue, cough, upper respiratory symptoms, myalgia, skin problems and chest problems than the elderly patients (Table 4). In contrast to younger patients, the elderly patients had more problems with level of consciousness, including drowsiness and confusion, and psychological issues such as anxiety and problems with isolation. There were also some differences in symptom manifestation between males and females in the descriptive comparison of studies on gender-specific studies (Table 5). Males experienced fever more frequently, whilst females more often had sore throat, muscle pain and diarrhoea.

**Table 4:** Themes and Issues by Age from observational studies, 305 papers

Themes and Issues	Young (48 papers)	Middle-aged (65 papers)	Elderly (19 papers)	Mixed (160 papers)	Young vs. elderly
	*n* (%)	*n* (%)	*n* (%)	*n* (%)	Difference in proportions
General	43 (90)	58 ( 89)	17 (89)	158 (99)	1
Fever	39 (81)	58 (89)	14 (74)	157 (98)	7
Fatigue or asthenia including weakness	18 (38)	26 (40)	5 (26)	103 (64)	12^*^
Level of consciousness	2 (4)	2 (3)	4 (21)	14 (9)	**–** 17^*^
Respiration	36 (75)	60 (92)	13 (68)	153 (96)	7
Cough	36 (75)	48 (74)	11 (58)	151 (94)	17*
Shortness of breath, dyspnoea or respiratory distress	22 (46)	37 (57)	8 (42)	126 (79)	4
Mouth/throat	12 (25)	13 (20)	2 (11)	93 (58)	14*
Pharyngodynia/sore throat	12 (25)	10 (15)	1 (5)	89 (56)	20*
Nose	13 (27)	8 (12)	0 (0)	60 (38)	27*
Ear/Eye	4 (8)	3 (5)	1 (5)	7 (4)	3
Pain	18 (38)	29 (45)	2 (11)	124 (78)	27*
Myalgia/general muscle pain	14 (29)	20 (31)	1 (5)	100 (63)	24*
Headache	4 (8)	15 (23)	0 (0)	96 (60)	8
Neuropathic- neurological	3 (6)	8 (12)	2 (11)	11 (7)	− 5
Gastrointestinal/Urinary	23 (48)	24 (37)	10 (53)	125 (78)	− 5
Diarrhoea	19 (40)	17 (26)	7 (37)	108 (68)	3
Skin	6 (13)	1 (2)	0 (0)	10 (6)	13*
Chest—cardio	14 (29)	12 (18)	3 (16)	49 (31)	13*
Psychological–psychiatric/concern/functions	3 (6)	8 (12)	5 (26)	20 (13)	-20*

Age not specified in 13 papers

*Absolute difference (young–elderly) in proportions > 10% shown in bold values

**Table 5:** Themes and Issues by gender from observational studies, 305 papers

Themes and Issues	Female (50 papers)	Male (65 papers)	Both gender (179 papers)	Female vs. male
	*n* (%)	*n* (%)	*n* (%)	Difference in proportions
General	41 (82)	61 (94)	176 (98)	− 12^*^
Fever	38 (76)	57 (88)	175 (98)	− 12^*^
Fatigue or asthenia including weakness	17 (34)	21 (32)	114 (64)	2
Level of consciousness	0 (0)	6 (9)	16 (9)	**− **9
Respiration	41 (82)	53 (82)	170 (95)	0
Cough	37 (74)	42 (65)	168 (94)	9
Shortness of breath, dyspnoea or respiratory distress	22 (44)	31 (48)	141 (79)	**− **4
Mouth/throat	11 (22)	4 (6)	105 (59)	16*
Pharyngodynia/sore throat	10 (20)	4 (6)	98 (55)	14*
Nose	5 (10)	11 (17)	65 (36)	**− **7
Ear/Eye	3 (6)	4 (6)	9 (5)	0
Pain	21 (42)	17 (26)	135 (75)	16*
Myalgia/general muscle pain	17 (34)	9 (14)	108 (60)	20*
Headache	5 (10)	8 (12)	102 (57)	**− **2
Neuropathic- neurological	5 (10)	4 (6)	15 (8)	4
Gastrointestinal/Urinary	23 (46)	25 (38)	133 (74)	8
Diarrhoea	20 (40)	16 (25)	114 (64)	15*
Skin	5 (10)	2 (3)	10 (6)	7
Chest—cardio	12 (24)	12 (18)	54 (30)	6
Psychological–psychiatric/concern/functions	0 (0)	5 (8)	10 (6)	**− **8

Gender unknown in 11 papers

*Absolute difference (female–male) in proportions > 10% shown in bold values

### Results of the updated literature search

In the updated search in October 2020 and February 2021, 74 of the 477 identified references met the inclusion criteria. Forty-five were observational studies, of which many included information on patients experiences of psychological symptoms (*n* = 30) and neurological symptoms (*n* = 15).

Sixteen studies provided results on long COVID, including persistent or late effects after discharge or during follow-up (online Appendix 7). The presence of different symptoms at different time points or over a certain time-period was reported, not the duration of each symptom. Across different continents, 28–74% of the patients had persistent respiratory symptoms and 28–72% had fatigue 1–4 months after discharge [23, 24], and this seemed to be independent of the severity of the COVID-19 infection [25]. Reduced physical and mental health were also observed in patients several weeks after discharge for severe COVID-19 [26]. Anxiety, depression, and cognitive deficits (affected short-term memory, attention and concentration) [24, 27, 28], pain, discomfort, sleep disturbances and headache were reported more than 4 weeks after discharge [27, 29]. In a longitudinal study, 538 survivors reported a large range of persistent symptoms 3 months after discharge (general, respiratory, cardiovascular and psychosocial) of which the most common were fatigue and hair loss [30]. The large symptom burden and reduced levels of functioning mean that many patients with COVID-19 experience reduced HRQoL for several months after infection [23, 31, 32]. Three new symptoms not included in Table 3 were identified: change of voice (*n* = 1) [32], dysphagia (*n* = 2) [32, 33] and suicidal thoughts (*n* = 1) [34]. In addition, one paper reported optimism, hope, resilience, and self-efficacy in nurses returning to work after recovering from the infection [35]. Also, PTSD and complications such as stroke and encephalopathy were described following COVID-19 in patients [34, 36, 37].

The updated search identified 24 publications that included PROMs. Most measures were symptom-specific (*n* = 20) measuring fatigue [25], respiratory symptoms [23] and mental health [37] and four studies used generic questionnaires: SF-36 [31, 38], EQ-5D [32] or Patient Health Questionnaire [23]. Twelve studies included more than two questionnaires. None of these questionnaires had been validated for use in patients with COVID-19.

## Discussion

This systematic literature review was performed with the aim of gathering as much information relevant to the HRQoL of patients with COVID-19 as possible. We therefore included all types of publications, study designs and research settings. The updated searches, performed 6 and 9 months later, identified virtually no new symptoms or concerns. This confirms that our review has succeeded in obtaining a comprehensive overview of HRQoL issues experienced by patients with current or previous symptomatic disease, and for the follow-up period. The content validity of the identified issues is therefore supported. Our review did not identify any PROM developed specifically for these patients, supporting the need for an internationally developed HRQoL instrument for patients during and after COVID-19. The multiplicity of devastating symptoms and functional deficit related to this disease, both in the acute phase and long-term follow-up may have large implications for the patients themselves and their families. COVID-19 affects people of all ages, and the symptom burden may also give reduced working abilities and societal challenges.

Despite COVID-19 being a novel disease, in this review, there was very good representation of patients from different care-settings and age groups, with several different comorbidities. The reports included both females and males, with the exception of some case reports and a few studies on gender-specific populations such as patients in a maternity ward [39]. Reports from China, the first country to publish descriptions on COVID-19-specific symptoms, focused on core symptoms such as fever, cough, shortness of breath and diarrhoea and a large proportion of patients across all ages needed hospital admission and intensive care. With time, as the disease spread and new phases of the disease trajectory were experienced, additional issues such as alteration of senses (taste, smell) [19] and cutaneous manifestations [40], began to appear as the complexity of the disease gradually came to light. This review includes studies from all continents; thereby reflecting cross-cultural HRQoL issues.

The large variation in type and severity of acute symptoms highlight the need for clinicians to have a high level of vigilance and initiate necessary measures when COVID-19 is suspected. For patients with confirmed diagnosis, all symptoms experienced and their influence on HRQoL need to be mapped, during active disease and in the follow-up period, in order to initiate adequate treatment and care. The COVID-19 HRQoL questionnaire developed in this project would be well suited for this purpose. The large variation in HRQoL issues that patients with COVID-19 experience during the infection and recovery period also highlights the need for cooperation between different levels of the health care services to ensure optimal care for patients along with the best possible use of resources.

The initial lack of psychological symptoms and concerns reported might have been caused by investigators who were focused on describing symptoms that could help identify and control the spread of the disease. At this stage, psychological symptoms, patient functioning, and concerns and how these issues impacted patients’ HRQoL might have been regarded as less important. The updated search revealed that more recent publications have had an increased focus on the psychological impact of the disease both during [41] and after discharge from hospital [24, 27, 42]. Patients in rehabilitation with post-acute COVID-19 had ongoing physical and psychological symptoms and reduced HRQoL [30, 32, 43]. Experiences from follow-up studies show that some patients with COVID-19 also experience fatigue, anxiety, depression, PTSD and impaired cognitive function in the long term [12, 28, 30, 44]. Most symptoms reported in the months after discharge seemed to be a continuation of symptoms experienced during active disease, but new symptoms were also described such as problems with attention, concentration, obsessive–compulsive symptoms and PTSD. This finding emphasises that COVID-19 entails a large variation in disease trajectories. Health care providers need to be aware of these differences in disease courses to be able to provide adequate care for patients during the acute phase of the disease and also during follow-up after COVID-19 infection.

Young patients reported more symptoms than elderly patients. This was contradictory to what we expected as elderly patients are at risk for developing more severe disease with rapid progression [6, 7]. A possible explanation is that elderly patients seeking medical care for other conditions were more often tested whereas young patients were only tested if they had the core symptoms. It was also interesting that elderly patients had more emotional symptoms than the young, counter to the usual finding that elderly people are less emotionally disturbed by illness [45]. Increased social isolation may have had more of an impact on older people, particularly because of the publicity around elderly people being at an increased risk of developing severe COVID-19. The possible differences in the acute symptom manifestation for males and females described in this review needs to be confirmed in other studies. Knowing more about gender differences would help clinicians better understand the disease. One study reported that males seemed to have a higher risk of getting severely ill than females and were more often in need of intensive care [46].

### Use of PROMs

Even though this disease carries a heavy symptom burden, the use of PROM remains rare. This may be partly explained by the lack of a COVID-19-specific PROM. Contrary to our expectations, only a few studies used generic questionnaires [31, 32, 38, 47]. Halpin et al., reported reduced HRQoL measured with the EQ-5D in more than 50% (53/100) of patients discharged from hospital after treatment for COVID-19 [32]. The problem with using generic questionnaires to assess HRQoL is their low sensitivity to change, along with the potential for weak content validity and missed symptoms. Therefore, it is often recommended to combine generic and disease-specific questionnaires [48]. Some studies used non-validated questionnaires [49, 50], which should be strongly discouraged as it entails a high risk for unreliable results.

This systematic literature review represents the first important step in the development of an international COVID-19-specific questionnaire, insofar as the results point to issues that should be included in the measure. Without such a questionnaire there is a significant risk that researchers might put together instruments that are incomplete and not validated for these patients. This can produce results that, in the worst case, could lead to misjudged treatment decisions and harm patients.

### Strengths and limitations

The strength of this comprehensive systematic literature review was the application of a robust methodology following international guidelines and the publishing of the protocol upfront in PROSPERO.

By including a variety of studies and publications, including letters and case reports without a systematic quality assessment, studies might have been biased, with non-representative patient populations. On the other hand, our focus was not to retrieve a quantitative assessment of the incidence/prevalence of symptoms, but rather to achieve a broad insight of these issues very early in the pandemic. Only English publications were reviewed, so issues reported in other languages might have been missed. We believe that the risk for this is small given the extensive list of issues identified and the very few additional issues identified in the updated review. Information on HRQoL issues specific to children is not covered in this review, even though some of the publications may include a broad age-range. Late effects more than a year after COVID-19 may be lacking, as the last literature search was performed less than 1.5 years after the start of the pandemic.

## Conclusion

In this comprehensive review we found a wide range of reported HRQoL issues affecting most of the organ systems in patients with COVID-19. The variety of clinical manifestations and their impact on patients’ HRQoL, together with the finding that all symptoms were present in the acute phase of the disease, is important information for clinicians to better recognize and understand the disease. This has important clinical implications for diagnosis, treatment Queryand follow-up of patients with COVID-19. For patients with COVID-19 and their next of kin, it can give greater insight into many different clinical aspects of the disease and support self-management. The results provided the foundation for the international development of a COVID-19 specific patient-reported HRQoL questionnaire.

## Supplementary Information

Below is the link to the electronic supplementary material.

Supplementary file1 (DOC 65 kb)

Supplementary file2 (DOC 63 kb)

Supplementary file3 (DOCX 18 kb)

Supplementary file4 (DOCX 17 kb)

Supplementary file5 (XLS 71 kb)

Supplementary file6 (XLS 357 kb)

Supplementary file7 (DOCX 27 kb)

## Data Availability

Not applicable.
