# Supplementary material for: Health-related quality of life issues, including symptoms, in patients with active COVID-19 or post COVID-19; a systematic literature review
Source: Qual Life Res. 2021 Jun 19;30(12):3367–81. doi: 10.1007/s11136-021-02908-z (PMC8214069; doi:10.1007/s11136-021-02908-z)
Supplement: Supplementary file 2 — Supplementary file2 (DOC 63 kb) [file 11136_2021_2908_MOESM2_ESM.doc]

SEARCH STRATEGIES MAIN SEARCH

|  | Database: Ovid MEDLINE(R) ALL <1946 to April 28, 2020>  Search Strategy: |
| --- | --- |
| 1  2  3  4  5  6  7  8  9  10 11  12  13  14 15  16  17  18  19  20  21 22  23  24 25  26  27  28  29  30  31  32  33 34  35  36 37 38 39 40  41 42 43 44  45  46  47 48  49 50 51  52 53 54  55 56  57 58  59 | Covid-19.tw,kf. (6423)  severe acute respiratory syndrome coronavirus 2.tw,kf. (620)  (exp Coronavirus infections/ or (corona virus or coronavirus).tw,kf.) and (Wuhan or Novel or Covid19 or Covid-19 or nCoV or CoV2 or nCov19 or SARS-CoV-2 or SARS-CoV2).tw,kf. (5971)  or/1-3 (8986)  adverse effects.fs. (1716620)  (side effect* or late effect* or long-term effect* or short-term effect*).tw,kf. (303907)  ((loss or lost or reduced or reduction or physical or emotional) adj3 function*).tw,kf. (133156)  exp Pain/ (391551)  Headache/ (27485)  Fever/ (38859)  Cough/ (15655)  exp pharyngitis/ (15556)  Rhinitis/ (12531)  Dyspnea/ (20895)  Respiratory Insufficiency/ (31752)  Ageusia/ (259)  Dysgeusia/ (413)  Olfaction disorders/ (4056)  Taste/ (22220)  Smell/ (15563)  Kidney/ or exp Kidney diseases/ (698808)  exp heart diseases/ or exp pneumonia/ (1203689)  exp diarrhea/ or exp vomiting/ (81103)  exp Gastrointestinal diseases/ (947880)  (respiratory adj3 (distress or discomfort or complication* or problem* or failure)).tw,kf. (77768)  ((breathing or breath or breathe) adj3 (difficult* or problem* or pain* or shortness)).tw,kf. (11224)  (fever or drowsiness or weakness or sleep deprivation or sleepiness or fatigue* or dizzy or dizziness).tw,kf. (352427)  (coughing or cough or muscle ache* or headache* or muscle pain).tw,kf. (135547)  (pain* adj3 (throat or tongue or head or eyes or ears or nose or mouth or oral or mucous membrane* or neck or shoulder* or joint or joints or muscle or stomach or chest or limbs or legs or arms or facial)).tw,kf. (85556)  (ageusia or dysgeusia or olfaction or olfactory or ((sense or function* or loss or alter*) adj3 (smell or smelling or taste or tasting))).tw,kf. (51101)  (kidney* or lung* or liver* or skin or heart or cardiovascular or cardiac or myocardial or pneumonia).tw,kf. (3847821)  (rapid heartbeat* or rapid heart rate or heart palpitations).tw,kf. (349)  (diarrhea* or vomiting* or nausea* or nauseous*).tw,kf. (157430)  (pharyngitis or sore throat or rhinitis stuffy nose or congested nose or throat infect*).tw,kf. (10988)  (gastrointestinal adj3 (infection* or disease* or symptom* or pain* or cramp*)).tw,kf. (35706)  Anxiety/ (79595)  Mental health/ (37342)  Fear/ (31235)  Psychological distress/ (365)  Stress, psychological/ (118955)  Depression/ (116847)  Psychology/ (23196)  social support/ or social isolation/ or quarantine/ (83785)  (anxious or anxiety or depression or depressed or fearful or fear or scared or psychological stress or psychological problems or psychological issues or psychological distress or hopelessness or hopefulness or mental state or mental health or loss of meaning).tw,kf. (697575)  (social support or social isolation or feeling of isolation or feeling alone or loneliness or quarantine*).tw,kf. (54374)  ((patient* adj3 experienc*) or (burden of illness or burden of disease or disease burden)).tw,kf. (156963)  Quality of life/ (191267)  exp Activities of daily living/ or (daily activities or activities of daily living).tw,kf. or ADL.ti,kw. (123649)  (quality of life or life quality).mp. (341086)  (HR-QOL or HR-PRO or HRPRO or HRQL or HRQoL or QL or QoL).tw,kf. (56995)  (wellbeing or well-being or sf-36 or life satisfaction or health index* or health indices or health profile* or prom or proms or pro-measure*).tw,kf. (122005)  exp Patient Reported Outcome Measures/ or exp patient satisfaction/ (93239)  patient outcome assessment/ or critical care outcomes/ (4604)  ((patient* or self* or carer) adj3 (outcome* or measure or appraisal* or appraised or report or reported or reporting or rated or rating* or based or assessed or assessment* or satisfaction)).tw,kf. (776671)  (patientreported or patientreporting or selfreported or selfreporting).tw,kf. (123)  or/5-55 (9013102)  4 and 56 (3946)  limit 57 to (english language and yr="2019 -Current") (3099)  58 use medall (3099) |

Database: Ovid MEDLINE(R) ALL <1946 to April 28, 2020>

Search Strategy:

--------------------------------------------------------------------------------

1 Covid-19.tw,kf. (6423)

2 severe acute respiratory syndrome coronavirus 2.tw,kf. (620)

3 (exp Coronavirus infections/ or (corona virus or coronavirus).tw,kf.) and (Wuhan or Novel or Covid19 or Covid-19 or nCoV or CoV2 or nCov19 or SARS-CoV-2 or SARS-CoV2).tw,kf. (5971)

4 or/1-3 (8986)

5 adverse effects.fs. (1716620)

6 (side effect* or late effect* or long-term effect* or short-term effect*).tw,kf. (303907)

7 ((loss or lost or reduced or reduction or physical or emotional) adj3 function*).tw,kf. (133156)

8 exp Pain/ (391551)

9 Headache/ (27485)

10 Fever/ (38859)

11 Cough/ (15655)

12 exp pharyngitis/ (15556)

13 Rhinitis/ (12531)

14 Dyspnea/ (20895)

15 Respiratory Insufficiency/ (31752)

16 Ageusia/ (259)

17 Dysgeusia/ (413)

18 Olfaction disorders/ (4056)

19 Taste/ (22220)

20 Smell/ (15563)

21 Kidney/ or exp Kidney diseases/ (698808)

22 exp heart diseases/ or exp pneumonia/ (1203689)

23 exp diarrhea/ or exp vomiting/ (81103)

24 exp Gastrointestinal diseases/ (947880)

25 (respiratory adj3 (distress or discomfort or complication* or problem* or failure)).tw,kf. (77768)

26 ((breathing or breath or breathe) adj3 (difficult* or problem* or pain* or shortness)).tw,kf. (11224)

27 (fever or drowsiness or weakness or sleep deprivation or sleepiness or fatigue* or dizzy or dizziness).tw,kf. (352427)

28 (coughing or cough or muscle ache* or headache* or muscle pain).tw,kf. (135547)

29 (pain* adj3 (throat or tongue or head or eyes or ears or nose or mouth or oral or mucous membrane* or neck or shoulder* or joint or joints or muscle or stomach or chest or limbs or legs or arms or facial)).tw,kf. (85556)

30 (ageusia or dysgeusia or olfaction or olfactory or ((sense or function* or loss or alter*) adj3 (smell or smelling or taste or tasting))).tw,kf. (51101)

31 (kidney* or lung* or liver* or skin or heart or cardiovascular or cardiac or myocardial or pneumonia).tw,kf. (3847821)

32 (rapid heartbeat* or rapid heart rate or heart palpitations).tw,kf. (349)

33 (diarrhea* or vomiting* or nausea* or nauseous*).tw,kf. (157430)

34 (pharyngitis or sore throat or rhinitis stuffy nose or congested nose or throat infect*).tw,kf. (10988)

35 (gastrointestinal adj3 (infection* or disease* or symptom* or pain* or cramp*)).tw,kf. (35706)

36 Anxiety/ (79595)

37 Mental health/ (37342)

38 Fear/ (31235)

39 Psychological distress/ (365)

40 Stress, psychological/ (118955)

41 Depression/ (116847)

42 Psychology/ (23196)

43 social support/ or social isolation/ or quarantine/ (83785)

44 (anxious or anxiety or depression or depressed or fearful or fear or scared or psychological stress or psychological problems or psychological issues or psychological distress or hopelessness or hopefulness or mental state or mental health or loss of meaning).tw,kf. (697575)

45 (social support or social isolation or feeling of isolation or feeling alone or loneliness or quarantine*).tw,kf. (54374)

46 ((patient* adj3 experienc*) or (burden of illness or burden of disease or disease burden)).tw,kf. (156963)

47 Quality of life/ (191267)

48 exp Activities of daily living/ or (daily activities or activities of daily living).tw,kf. or ADL.ti,kw. (123649)

49 (quality of life or life quality).mp. (341086)

50 (HR-QOL or HR-PRO or HRPRO or HRQL or HRQoL or QL or QoL).tw,kf. (56995)

51 (wellbeing or well-being or sf-36 or life satisfaction or health index* or health indices or health profile* or prom or proms or pro-measure*).tw,kf. (122005)

52 exp Patient Reported Outcome Measures/ or exp patient satisfaction/ (93239)

53 patient outcome assessment/ or critical care outcomes/ (4604)

54 ((patient* or self* or carer) adj3 (outcome* or measure or appraisal* or appraised or report or reported or reporting or rated or rating* or based or assessed or assessment* or satisfaction)).tw,kf. (776671)

55 (patientreported or patientreporting or selfreported or selfreporting).tw,kf. (123)

56 or/5-55 (9013102)

57 4 and 56 (3946)

58 limit 57 to (english language and yr="2019 -Current") (3099)

59 58 use medall (3099)

***************************

Database: Embase Classic+Embase <1947 to 2020 April 27>

Search Strategy:

--------------------------------------------------------------------------------

1 "coronavirus disease 2019"/ (1082)

2 (exp coronaviridae infection/ or exp Coronaviridae/ or (coronavirus or corona virus).tw,kw.) and (Wuhan or Novel or Covid19 or Covid-19 or nCoV or CoV2 or nCov19 or SARS-CoV-2 or SARS-CoV2).tw,kw. (5726)

3 (covid-19 or covid19 or severe acute respiratory syndrome coronavirus 2).tw,kw. (4578)

4 1 or 2 or 3 (7795)

5 (side effect* or late effect* or long-term effect* or short-term effect*).tw,kw. (457378)

6 ((loss or lost or reduced or reduction or physical or emotional) adj3 function*).tw,kw. (193142)

7 pain/ (341119)

8 headache/ (222309)

9 fever/ (251619)

10 pneumonia/ (182672)

11 coughing/ (109892)

12 exp pharyngitis/ (32210)

13 exp *rhinitis/ (38752)

14 exp breathing disorder/ (337898)

15 exp respiratory failure/ (105803)

16 exp taste disorder/ (15565)

17 exp smelling disorder/ (10676)

18 exp sensory dysfunction/ (614612)

19 exp "smelling and taste"/ (76974)

20 exp kidney disease/ or exp kidney/ (1311752)

21 exp heart disease/ (1973481)

22 exp diarrhea/ (265324)

23 exp vomiting/ (216036)

24 gastrointestinal disease/ (94008)

25 (respiratory adj3 (distress or discomfort or complication* or problem* or failure)).tw,kw. (125641)

26 ((breathing or breath or breathe) adj3 (difficult* or problem* or pain* or shortness)).tw,kw. (24163)

27 (fever or drowsiness or weakness or sleep deprivation or sleepiness or fatigue* or dizzy or dizziness).tw,kw. (555316)

28 (pneumonia or coughing or cough or muscle ache* or headache* or muscle pain).tw,kw. (400095)

29 ((discomfort* or pain*) adj3 (throat or tongue or head or eyes or ears or nose or mouth or oral or mucous membrane* or neck or shoulder* or joint or joints or muscle or stomach or chest or limbs or legs or arms or facial)).tw,kw. (145228)

30 (ageusia or dysgeusia or olfaction or olfactory or ((sense or function* or loss or alter*) adj3 (smell or smelling or taste or tasting))).tw,kw. (66079)

31 (kidney* or lung* or liver* or skin or heart or cardiovascular or cardiac or myocardial).tw,kw. (5467756)

32 (rapid heartbeat* or rapid heart rate or heart palpitations).tw,kw. (570)

33 (diarrhea* or vomiting* or nausea* or nauseous*).tw,kw. (260273)

34 (pharyngitis or rhinitis or sore throat or stuffy nose or throat infect*).tw,kw. (60151)

35 (gastrointestinal adj3 (infection* or disease* or symptom* or pain* or cramp*)).tw,kw. (52211)

36 exp fear/ (269644)

37 mental health/ or psychological well-being/ (155016)

38 distress syndrome/ (42689)

39 mental stress/ (84167)

40 depression/ (367958)

41 anxiety disorder/ (69992)

42 psychology/ (180891)

43 social support/ (89754)

44 exp social isolation/ (23851)

45 quarantine/ (951)

46 (anxious or anxiety or depression or depressed or fearful or fear or scared or psychological stress or psychological problems or psychological issues or psychological distress or hopelessness or hopefulness or mental state or mental health or loss of meaning).tw,kw. (971449)

47 (social support or psychological support or social isolation or feeling of isolation or feeling alone or loneliness or quarantine*).tw,kw. (75106)

48 ((patient* adj3 experienc*) or (burden of illness or burden of disease or disease burden)).tw,kw. (255883)

49 exp "quality of life"/ (482972)

50 daily life activity/ (88888)

51 (quality of life or life quality).mp. (588473)

52 (HR-QOL or HR-PRO or HRPRO or HRQL or HRQoL or QL or QoL).tw,kw. (102859)

53 (wellbeing or well-being or sf-36 or life satisfaction or health index* or health indices or health profile* or prom or proms or pro-measure*).tw,kw. (169059)

54 patient-reported outcome/ (21445)

55 patient satisfaction/ (136209)

56 ((patient* or self* or carer) adj3 (outcome* or measure or appraisal* or appraised or report or reported or reporting or rated or rating* or based or assessed or assessment* or satisfaction)).tw,kw. (1215595)

57 or/5-56 (11357311)

58 4 and 57 (2944)

59 limit 58 to (english and yr="2019 -Current") (2067)

60 59 use emczd (2067)

***************************

Database: APA PsycInfo <1806 to April Week 3 2020>

Search Strategy:

--------------------------------------------------------------------------------

1 (covid-19 or covid19 or severe acute respiratory syndrome coronavirus 2).mp. (9)

2 ((corona virus or coronavirus) and (Wuhan or Novel or Covid19 or Covid-19 or nCoV or CoV2 or nCov19 or SARS-CoV-2 or SARS-CoV2)).mp. (17)

3 1 or 2 (22)

4 limit 3 to (english and yr="2019 -Current") (6)

***************************

Cinahl search strategy

| **#** | **Query** | **Results** |
| --- | --- | --- |
| S1 | (MH "Coronavirus+") OR (MH "Coronavirus Infections+") | 3,438 |
| S2 | corona virus or coronavirus | 2,251 |
| S3 | S1 OR S2 | 4,129 |
| S4 | TX (Wuhan or Novel or Covid19 or Covid-19 or nCoV or CoV2 or nCov19 or SARS-CoV-2 or SARS-CoV2) | 133,777 |
| S5 | S3 AND S4 | 987 |
| S6 | (MH "Quality of Life+") OR (MH "Quality-Adjusted Life Years") | 128,988 |
| S7 | quality of life or well being or well-being or health-related quality of life | 0 |
| S8 | quality of life or well being or well-being or health-related quality of life | 250,002 |
| S9 | (MH "Patient-Reported Outcomes") | 2,277 |
| S10 | headache or migraine or pain or muscle ache | 352,102 |
| S11 | ( sore throat or tonsilitis or throat or strep throat ) OR ( pharyngitis or rhinitis or throat infection ) OR ( stuffed nose or congested nose or hoarseness ) | 17,733 |
| S12 | ( (sleepiness or drowsiness or fatigue or dizzy or dizziness or sleeplessness or sleepdeprivation) ) OR ( (liver or lung* or heart* or cardiac or cardiovascular or myocardial) ) OR ( gastrointestinal NEAR2 (pain* or problem* or disorder* or cramp* or symptom*) ) | 775,560 |
| S13 | (MH "Fever+") | 12,142 |
| S14 | (MH "Pneumonia+") | 22,968 |
| S15 | (MH "Anxiety+") | 48,338 |
| S16 | (MH "Depression+") | 119,992 |
| S17 | (MH "Fear+") | 14,824 |
| S18 | social isolation or quarantine or social support or psychological support or loneliness | 5,724 |
| S19 | S6 OR S7 OR S8 OR S9 OR S10 OR S11 OR S12 OR S13 OR S14 OR S15 OR S16 OR S17 OR S18 | 1,438,982 |
| S20 | S5 AND S19 | 374 |
| S21 | S5 AND S19 | 45 |
|  | Limiters - Published Date: 20190101-20201231; Peer Reviewed; Exclude MEDLINE records; Language: English  Expanders - Apply equivalent subjects  Search modes - Boolean/Phrase |  |
