# Supplementary material for: Health-related quality of life issues, including symptoms, in patients with active COVID-19 or post COVID-19; a systematic literature review
Source: Qual Life Res. 2021 Jun 19;30(12):3367–81. doi: 10.1007/s11136-021-02908-z (PMC8214069; doi:10.1007/s11136-021-02908-z)
Supplement: Supplementary file 3 — Supplementary file3 (DOCX 18 kb) [file 11136_2021_2908_MOESM3_ESM.docx]

**Appendix 3 Checklist 93 criteria for data extraction**

| **Nr** | **Criteria** |
| --- | --- |
|  | **Inclusion criteria** |
| 1 | Verified COVID-19 |
| 2 | Empirical reports symptoms, functions, concerns |
|  | **Exclusion criteria** |
| 3 | Not English |
| 4 | Children (< age of 18 years) only |
| 5 | Experiences related to the COVID-19 situation in the society |
| 6 | basal biology, screening, or diagnostics |
| 7 | Experiences specifically related to ICU-treatment |
| 8 | Experiences related to other disease groups |
| 9 | Other experience with the pandemic issue not related phenomenon |
|  | **general information on the paper and patient characteristics** |
| 10 | Country of patients |
| 11 | Type of study |
| 12 | Type of report |
| 13 | Type of PROM |
| 14 | Number of patients |
| 15 | Gender |
| 16 | Age specified |
| 17 | Co-morbidity |
| 18 | Hospitalisation |
|  | **Reported patient experienced COVID-19 HRQoL issues** |
| 19 | Fever |
| 20 | Chills |
| 21 | Fatigue/Asthenia |
| 22 | Malaise/ Feeling sick |
| 23 | Extensive sweating |
| 24 | Dizziness |
| 25 | Drowsiness |
| 26 | Confusion (Delirium) |
| 27 | Unconscious |
| 28 | Cough |
| 29 | Sneezing |
| 30 | Shortness of breath/Dyspnea/Respiratory distress |
| 31 | Expectoration lung |
| 32 | Hemoptysis |
| 33 | Oral (mouth) Mucus/saliva |
| 35 | Tonsil swelling |
| 36 | Throat congestion |
| 37 | Mucus nose/Nasal congestion |
| 38 | Runny nose/ Rhinorrhea/coryza |
| 39 | Sore throat/ pharyngodynia |
| 40 | Otalgi/ Ear pain |
| 41 | Hearing loss |
| 42 | Irritation/ Sore eyes |
| 43 | Red eyes |
| 44 | Pain/ general pain |
| 45 | Myalgia/general muscle pain |
| 46 | Neurological pain |
| 47 | Chestpain |
| 48 | Headache |
| 49 | Dysuria |
| 50 | Loss of taste |
| 51 | Loss of smell |
| 53 | Vision |
| 54 | Uncoordinated movments/ ataxi |
| 55 | Seizure/ cramps |
| 56 | Diarrhea |
| 57 | Constipation |
| 58 | Nausea |
| 59 | Vomiting |
| 60 | Anorexia |
| 61 | Abdominal pain/ Stomachache |
| 62 | Rash |
| 63 | pruritus |
| 64 | Burning sensation/ skin pain/ hypersensitivity |
| 65 | Hair loss |
| 66 | Tightness of chest |
| 67 | Chest distress |
| 68 | Heart palpitations |
| 69 | Other symptoms |
| 70 | Physical functioning |
| 71 | Activity of daily living, eat, sleap, dress… |
| 72 | perform work/housekeeping |
| 73 | perform hobbies/leisure activities |
| 74 | Anxiety |
| 75 | Depression |
| 76 | Distress |
| 77 | Tension |
| 78 | Agitation |
| 79 | Anger |
| 80 | Concentration |
| 81 | Memory |
| 82 | social activities |
| 83 | other functioning issues |
| 84 | Fear of infecting others |
| 85 | Shame/guilt of having infected others |
| 86 | Fear of being discriminated against society |
| 87 | fear of future outcome |
| 88 | Fear of oeconomical consequences |
| 89 | Fear of cold |
| 90 | other concern Describe and classify |
| 91 | Overall health |
| 92 | Overall health- related quality of life |
|  | **PROM** |
| 93 | If PROM included, details type of Questionnaire |
