# Supplementary material for: Health-related quality of life issues, including symptoms, in patients with active COVID-19 or post COVID-19; a systematic literature review
Source: Qual Life Res. 2021 Jun 19;30(12):3367–81. doi: 10.1007/s11136-021-02908-z (PMC8214069; doi:10.1007/s11136-021-02908-z)
Supplement: Supplementary file 4 — Supplementary file4 (DOCX 17 kb) [file 11136_2021_2908_MOESM4_ESM.docx]

|  | Database: Ovid MEDLINE(R) ALL <1946 to October 29, 2020>  Search Strategy: |
| --- | --- |
| 1  2  3  4 5  6 7  8  9  10  11  12  13  14  15  16  17  18  19  20  21  22  23  24  25  26  27  28  29  30  31  32      33  34  35  36 | Covid-19.tw,kf. (51161)  severe acute respiratory syndrome coronavirus 2.tw,kf. (5550)  (exp Coronavirus infections/ or (corona virus or coronavirus).tw,kf.) and (Wuhan or Novel or Covid19 or Covid-19  or nCoV or CoV2 or nCov19 or SARS-CoV-2 or SARS-CoV2).tw,kf. (38855)  1 or 2 or 3 (55990)  anxiety/ or catastrophization/ (83091)  Mental Health/ (39250)  Fear/ (32000)  Psychological Distress/ (824)  Stress, Psychological/ (121328)  Depression/ (120481)  psychology/ (23406)  Mental disorders/ (163343)  ((loss or lost or reduced or reduction) adj3 function*).tw,kf. (96397)  ((physical or psychological or emotional or cognitive or mental or behavioral or functional) adj3 impairment*).tw,kf. (101264)  ((emotional or psychological or cognitive or behavior* or mental or physical) adj3 symptom*).tw,kf. (51405)  (anxious or anxiety or depression or depressed or fearful or fear or scared or psychological stress or psychological problems or psychological issues or psychological distress or hopelessness or hopefulness or mental state  or mental health or loss of meaning).tw,kf. (729483)  or/5-16 (1183803)  4 and 17 (3605)  Quality of life/ (197470)  (quality or life or life quality).tw,kf. (1796588)  (HR-QOL or HR-PRO or HRPRO or HRQL or HRQoL or QL or QoL or wellbeing or well-being or sf-36 or life satisfaction).tw,kf. (168224)  exp Patient Reported Outcome Measures/ (6454)  patient outcome assessment/ (4756)  ((patient* or self* or carer) adj3 (outcome* or measure or appraisal* or appraised or report or reported or  reporting or rated or rating* or based or assessed or assessment* or satisfaction)).tw,kf. (819548)  (patientreported or patientreporting or selfreported or selfreporting or patient experiences).tw,kf. (2784)  or/19-25 (2565172)  18 and 26 (1027)  limit 27 to english (990)  remove duplicates from 28 (987)  limit 29 to dt=20200410-20201231 (960)  exp Patients/ (65915)  (covid-19 patient* or covid-19 infected or covid-19 infection* or covid-19 survivor* or covid-19 positive or covid patient* or Cov2-patient* or Cov2-infected* or Cov2-infection* or Cov2-survior* or Cov2-positive or covid infected or covid infection* or covid survivor* or covid positive or corona* patient* or corona* infected or corona* infection*  or corona* survivor* or corona* positive).tw,kf. (12971)  ((infected or positive or patient*) adj3 (corona* or covid* or Wuhan or Covid19 or Covid-19 or nCoV or CoV2 or nCov19 or SARS-CoV-2 or SARS-CoV2)).tw,kf. (93363)  ((infected or infection) adj2 patient*).tw,kf. (87039)  31 or 32 or 33 or 34 (247320)  30 and 35 (238) |

Database: Ovid MEDLINE(R) ALL <1946 to January 29, 2021>

Search Strategy:

--------------------------------------------------------------------------------

1 (Covid-19 or covid19).tw,kf. (87335)

2 severe acute respiratory syndrome coronavirus 2.tw,kf. (10027)

3 exp Coronavirus infections/ or (corona virus or coronavirus).tw,kf. (79656)

4 (Wuhan or Novel or Covid19 or Covid-19 or nCoV or CoV2 or nCov19 or SARS-CoV-2 or SARS-CoV2).tw,kf. (1427605)

5 3 and 4 (61229)

6 1 or 2 or 5 (93676)

7 (late adj3 effect*).tw,kw. (12299)

8 (lateeffect* or aftereffect* or after effect*).tw,kw. (6677)

9 ((complication* or symptom* or health problem* or adverse effect* or health status or impairment*) adj4 (after

recovery or after discharge* or post-covid or post-discharge or postdischarge or postrecovery or post-recovery)).tw,kw.

(1108)

10 sequela.tw,kf. (5999)

11 (following adj2 (Covid* or nCoV or CoV2 or nCov19 or SARS-CoV-2 or SARS-CoV2 or severe acute respiratory syndrome

coronavirus 2 or coronavirus) adj2 (recovery or infection* or illness* or disease* or convalescence)).tw,kf. (233)

12 or/7-11 (26253)

13 exp Patients/ (67885)

14 (covid-19 patient* or covid-19 infected or covid-19 infection* or covid-19 survivor* or covid-19 positive or

covid patient* or Cov2-patient* or Cov2-infected* or Cov2-infection* or Cov2-survior* or Cov2-positive or covid infected

or covid infection* or covid survivor* or covid positive or corona* patient* or corona* infected or corona* infection*

or corona* survivor* or corona* positive).tw,kf. (20474)

15 ((infected or positive or patient*) adj3 (corona* or covid* or Wuhan or Covid19 or Covid-19 or nCoV or CoV2 or

nCov19 or SARS-CoV-2 or SARS-CoV2)).tw,kf. (104579)

16 ((infected or infection) adj2 patient*).tw,kf. (88268)

17 13 or 14 or 15 or 16 (262255)

18 6 and 12 and 17 (201)

19 from 18 keep 1-201 (201)

20 limit 19 to english (193)

21 remove duplicates from 20 (184)
