# Supplementary material for: Health-related quality of life issues, including symptoms, in patients with active COVID-19 or post COVID-19; a systematic literature review
Source: Qual Life Res. 2021 Jun 19;30(12):3367–81. doi: 10.1007/s11136-021-02908-z (PMC8214069; doi:10.1007/s11136-021-02908-z)
Supplement: Supplementary file 7 — Supplementary file7 (DOCX 27 kb) [file 11136_2021_2908_MOESM7_ESM.docx]

**Appendix 7 Overview of COVID-19 studies with patient-reported assessment of post recovery symptoms and functioning**

| **First author, 2020** | **Title** | **Country** | **Type of study** | **PROM used** | **Number of patients / characteristics** | **Time of assessment** | **Symptoms, functioning and concerns in %) for studies with N ≥ 50**  **in bold numbers ( n) for studies with N < 50** | |
| --- | --- | --- | --- | --- | --- | --- | --- | --- |
| Akter F | Clinical characteristics and short term outcomes after recovery from COVID-19 in patients with and without diabetes in Bangladesh | Bangladesh | Cross-sectional study | Interview | 734 /Diabetics | 4 weeks after negative test | Moderate pain/discomfort  Reduced mobility  Problems with self-care  Anxiety or depression  Sleep disturbances  Loss of concentration  Reduced memory  Hair loss | 30  17  10  22  30  26  19  10 |
| Cai X | Psychological Distress and Its Correlates Among COVID-19 Survivors During Early Convalescence Across Age Groups | China | Cross-sectional  study | Posttraumatic stress disorder self-rating scale (PTSD-SS),  Self-rating depression scale (SDS)  Self-rating anxiety scale (SAS) | 126 /mixed severity of COVID-19 | 14 days after discharge | Anxiety  Depression  Stress response | 38  22  31 |
| Chen KY | Predictors of Health-Related Quality of Life and Influencing Factors for COVID-19 Patients, a Follow-Up at One Month | China | Cross-sectional study | SF-36 | 504 /mixed severity of COVID-19 | 1 month after discharge | Reduced mental health  Reduced physical functioning  Reduced role functioning  Social functioning Emotional functioning  compared to norm.data.  Elderly most affected. | 49  16 |
| Daher A | Follow up of patients with severe coronavirus disease 2019 (COVID-19): Pulmonary and extrapulmonary disease sequelae | Germany | Longitudinal study | Patient Health Questionnaire 9 (PHQ-9) of depression  Generalized Anxiety Disorder 7 (GAD-7)  St. George’s Respiratory  Questionnaire (SGRQ)  EQ-5D | 33 /with severe disease | 6 weeks after discharge | Fatigue  Dyspnoea  Cough  Runny nose  Chest pain  Myalgia  Headache  Cognitive disorder  Loss of smell  Loss of taste  Gastrointestinal symptoms | **15**  **11**  **11**  **4**  **6**  **5**  **5**  **6**  **4**  **3**  **3** |
| Halpin SJ | Postdischarge symptoms and rehabilitation needs in survivors of COVID-19 infection: A cross-sectional evaluation | UK | Cross sectional study | Telephone screening tool developed for this study  EQ-5D | 100 /mixed severity of COVID-19 | 29-71 days (mean 48 days) after discharge | Fatigue  Breathlessness  Problems with speech  Worsened mobility  Reduced activities  PTSD  Anxiety and depression | 64  50  20  37  44  31  23 |
| Kamal M | Assessment and characterisation of post-COVID-19 manifestations | Egypt | Cross sectional study | Self-reported questionnaire (no information on validation) | 287 /mixed severity of COVID-19 | not specified "after recovery" | Fatigue  Continuous headache  Dyspnoea  Chest pain  Intermittent fever  Joint pain  Blurred vision  Tinnitus  Dementia  Anxiety  Depression  Obsessive-compulsory disorder | 72  29  28  29  11  31  17  17  29  38  29  5 |
| Liu K | Respiratory rehabilitation in elderly patients with COVID-19: A randomized controlled study | China | Prospective quasi-experiemental | SF-36  Self-rating depression scale (SDS)  Self-rating anxiety scale (SAS) | 72/  elderly patients | 6 weeks after discharge | Elderly patients on exercise program had improved physical, role, social and emotional functioning compared to controls | na |
| Mizrahi, B. | Longitudinal symptom dynamics of COVID-19 infection | Israel | Longitudinal | Self-reported symptom survey (no information on validation) | 278 /mixed severity of COVID-19 | 6 weeks after recovery | Fatigue  Shortness of breath  Runny nose  Myalgia | na  na  na  na |
| Mazza M | Anxiety and depression in COVID-19 survivors: Role of inflammatory and clinical predictors | Italy | Cross-sectional study | Multiple questionnaires  Impact of Events Scale-Revised (IES-R), PTSD Checklist for DSM-5 (PCL-5), Zung Self-Rating Depression Scale (ZSDS), 13-item Beck’s  Depression Inventory (BDI-13) and others | 402, /mixed severity of COVID-19 | 1 month after discharge | Depression  Anxiety  Insomnia  Obsessive-compulsive symptoms  PTSD | 31  42  40  20  28 |
| Negrini F | Neuropsychological Features of Severe Hospitalized Coronavirus Disease 2019 Patients at Clinical Stability and Clues for Postacute Rehabilitation | Italy | Cross-sectional study | Observer rated  Mini-Mental State Examination (MMSE),5 which is a general  cognitive assessment, and the Frontal Assessment Battery, | 9 /mixed severity of COVID-19 | 1 month after diagnosis | Cognitive delay  Anxiety  Depression | **3**  **6**  **2** |
| Townsend, L. | Persistent fatigue following SARS-CoV-2 infection is common and independent of severity of initial infection | Irland | Cross sectional | Chalders Fatigue questionnaire | 128 /mixed severity of COVID-19 | 6 weeks after recovery or discharge | Fatigue | 52 |
| Weerahandi, H. | Post-Discharge Health Status and Symptoms in Patients with Severe COVID-19 | USA | Observational cohort study | PROMIS Global health  PROMIS Dyspnea characteristics | 161/  severe COVID-19 | 1 month after discharge | Shortness of breath  Reduced physical and mental health compared to pre COVID | 74  na  na |
| Woo MS | Frequent neurocognitive deficits after recovery from mild COVID-19 | China | Cross sectional | Modified Telephone Interviews for Cognitive status  Personal Health Questionniare  Fatigue Assessment scale | 18/ mild to moderate COVID-19 | 20-105 days after recovery | Fatigue  Attention deficit  Concentration deficit  Short term memory deficit  Trouble finding words Severe mood swings | **2**  **9**  **8**  **5**  **3**  **1** |
| Xiong Q | Clinical sequelae of COVID-19 survivors in Wuhan, China: a single-centre longitudinal study | China | Longitudinal | Telephone follow up survey | 532 /mixed severity of COVID-19 | 91-116 days after discharge (3-4 month) | Fatigue  General symptoms  Arthralgia  Respiratory symptoms  Chest pain  Cardiovascular symptoms  Psychosocial symptoms  Alopecia | 28  50  8  39  12  13  23  29 |
| Yuan B | Correlation between immune response and self-reported depression during convalescence from COVID-19 | China | Cross-sectional | Self-rating depression scale (SDS) | 96 /mixed severity of COVID-19 | Early recovery, just after discharge not specified in more detail | Depression | 44 |
| Zheng N | Investigation of the Status of Nurses Returning to Work After Recovering From COVID-19 and Influencing Factors | China | Cross sectional survey | The Work Adaptation Scale  The Psychological Capital Scale | 75/ nurses | 14-72 days after discharge | Difficult to adapt to work  Poor ability to cope with stress | |
